# Pharmacogenetics and Psychiatric Care: A Review and Commentary

**Published:** 2018-04-16

**Authors:** Merlin G. Butler

**Affiliations:** * Departments of Psychiatry & Behavioral Sciences and Pediatrics, University of Kansas Medical Center, Kansas City, Kansas, USA

## Introduction

Personalized or precision medicine is emerging in the treatment of human diseases and management based on each individual’s genetic pattern and response to drugs categorized into two areas: 1) pharmacogenetics and 2) pharmacogenomics^1–5^. Pharmacogenetics is the study of DNA structural variations and impact on drug metabolism, efficacy and tolerability. DNA remains stable and does not change with time or age. Pharmacogenetics is most often based on the cytochrome P450 enzyme system, primarily found in the liver and involves genes coding for the production of cytochrome P450 enzymes^6–9^. The response to medications depends on each individual’s ability to metabolize drugs with most drugs broken down by this enzyme system dependent on the genetic makeup of each person. Pharmacogenomics is the study of DNA and RNA characteristics impacting gene function but can change or be influenced by factors (e.g., environment)^10–12^. Hence, pharmacogenetics deals with single genes and their structure while pharmacogenomics relates to gene function influenced by the environment, both can play a role in human disease including drug metabolism. The Federal Drug Administration (FDA) and National Institutes of Health (NIH) have identified pharmacogenetics and pharmacogenomics as important key tools in development and testing of new drugs and their impact in treating individuals with human disease^10,13,14^.

Inter-individual variability in drug response is now recognized as a major clinical problem in westernized societies where polymedication is common. Relevant gene polymorphisms with different racial distributions are identified sources of variability in drug responses by the modulation of drug-metabolizing cytochrome P450 enzymes discussed in this report. The conceptualization of drug interaction and potential relationship to an individual’s ability to break down drugs or metabolism influenced by genetics was raised by Motulsky in 1957^15,16^ and later supported by genetic-based pharmacokinetics research. For several years, it was known that certain anesthetic agents and doses would be altered depending on the individual’s response, signs and symptoms during surgical procedures when anesthesia was administered indicating variable responses from person to person possibly related to differences in their genetic patterns.

## Background and Description of Cytochrome P450 Enzyme System

Cytochrome P450 hepatic enzymes, primarily found in the mitochondrial inner-membrane or endoplasmic reticulum in cells are encoded by protein-coding genes located throughout the genome with over 50 in number^7^. This enzyme system includes a superfamily of heme-thiolate proteins with a special absorbance peak at 450 nm and named accordingly. The cytochrome P450 gene is thought to have originated about 3 billion years ago with repeated duplications leading to this multi-gene family and represents one of the largest family of genes in humans^9^.

Cytochrome P450 enzymes metabolize endogenous and xenobiotic substrates including environmental pollutants, agro-and plant chemicals but also play a role in biosynthesis and metabolism of steroids, lipids, vitamins, hormones and prostaglandins and also central in drug metabolism in humans^2,9^. However, approximately 90% of all drugs are metabolized by just seven different cytochrome enzymes including *CYP1A2, CYP3A4, CYP3A5, CYPC19, CYP2D6, CYP2C9* and *CYP2B6*^9,17,18^. For example, *CYP2D6* by itself contributes to the breakdown or oxidative metabolism of 25% of most commonly prescribed medications including tricyclic anti-depressants, opioids, anti-psychotics, tamoxifen, cough suppressants and anti-arrhythmics accounting for side effects in a large percentage of the general population if the individual is predisposed by specific *CYP2D6* gene variants now detectable by pharmacogenetics testing^8,19–25^.

**Figure 1.**
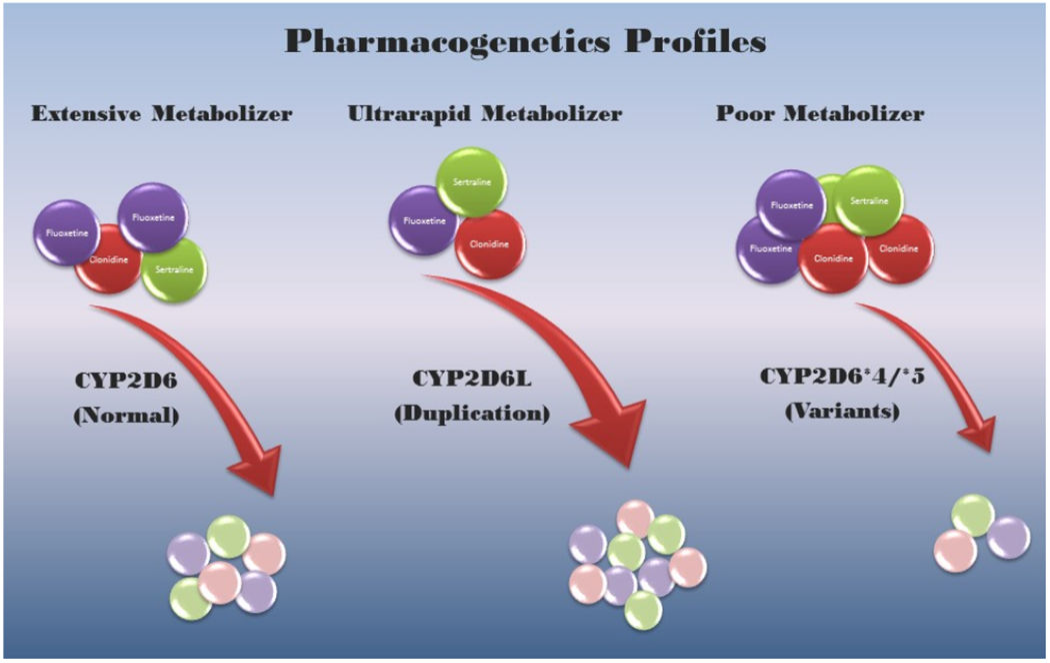
A schematic view of pharmacogenetics and drug profiles representing normal, ultrarapid and poor metabolizers of drugs for specific *CYP2D6* genotypes corresponding to each metabolic pattern. More breakdown products of representative drugs are generated by the *CYP2D6* duplication genotype with excessive enzyme activity and fewer breakdown products generated by the poor metabolism genotype as illustrated.

The *CYP2D6* gene is located on chromosome 22ql3.1 and consists of nine exons coding for 497 amino acids of this cytochrome P450 enzyme^9,20,21^. This enzyme has high affinity as an oxidase but accounts for less than 5% of all cytochrome P450 enzymes produced^9,26–28^. This gene is highly polymorphic with over 130 genetic variants with disturbances including duplications, single nucleotide polymorphisms (SNPs), splice defects, deletions and frame shift mutations. For example, a patient carrying *CYP2D6* *4/*5 genotype shows significant reduction in enzyme activity. The *5 variant represents a deletion of the second allele while the first allele (*4) is due to a G to A transition in the first nucleotide of exon 4; hence, both alleles show disturbances^20,21,29^ (see Figure 1).

SNPs can lead to decreased, increased or nonfunctional enzyme activity and impact the promoter of the gene regulating expression. Deletions account for no or poor enzyme activity, while duplications/multiplications of the functional or wild-type allele account for increased or rapid activity. Drugs that are metabolized by cytochrome P450 enzymes often have more than one enzyme involved in their breakdown. In addition, some drugs require metabolism to generate an active metabolite or functional therapeutic agent (see Table 1).

## Drug-Drug Interaction, Inducers and Inhibitors

Evidence now indicates that cytochrome P450 enzymes may be altered by the environment in the form of inhibitors or inducers as well as impacted by drug-drug interactions. Known inhibitors or inducers may include common food sources such as grapefruit, broccoli, cauliflower, garlic and coffee (caffeine) that alter enzyme activity by inhibiting or inducing the enzyme response and therefore affect the drug level. If an individual has the rapid form of the cytochrome P450 enzyme, then an inhibitor of that enzyme may lower the enzyme response in breaking down the drug influencing the response for that person and efficacy. Other prescribed drugs with drug-drug interaction can also act as inducers or inhibitors at the time the drug of interest is consumed and should be considered when prescribing new drugs. The impact of co-medication depends on drug to drug interaction, concentration of the inhibitor or inducer and their half-life with importance of the metabolic pathway. Once the inhibitor or inducer is removed from the environment then the cytochrome P450 enzyme can return to a normal activity if the cytochrome P450 gene is functional or wild-type in nature. Variation in sensitivity and enzyme action can contribute to drug response and side effects, directly relevant to medical management and drug selection, dosage and class. For example, about one in every 15 individuals will show an altered response to doses of beta blockers, a class of medication often prescribed for hypertension and metabolized by the cytochrome P450 enzyme system^17,30^. If this enzyme system is disturbed due to gene variants then side effects may occur that are harmful to the patient. Other agents affecting drug metabolism include smoking, glucocorticoids, progesterone, St. John’s wort and cough medicine^8^ (see Table 2).

## Pharmacogenetics-based Drug Dosing Guidelines in Psychiatry

Personalized medicine requires development of resources for clinicians and pharmacogenetic dosing guidelines for medications as 25-50% of individuals do not respond normally to drug treatment and dosage^31^. There are multiple guidelines in various stages of development during the past 5 years including those by the Clinical Pharmacogenetics Implementation Consortium (CPIC), a registered service mark of the US Department of Health and Human Services and other authoritative scientific consortia [(e.g., Dutch Pharmacogenetic Working Group (DPWG)] for cytochrome P450 and drug therapy including for psychiatry^32,33^. Other published reviews cite evidence and resources to implement pharmacogenetics testing as 90% of patients who undergo this testing are expected to have a clinically actionable result^34,35^. These reviews on cytochrome P450 and drug therapy should be examined by clinicians when prescribing drugs. Also, integration of pharmacogenetics data and gene variants from patients into electronic health records and access for clinical decision support will become more important, particularly in psychiatry where patients are often treated with multiple medications^36^.

Guidelines in psychiatry related to pharmacogenetics are growing with over 40 references found in the medical literature when searching PubMed (www.ncbi.nlm.nih.gov) for CPIC guidelines and 8 found when adding psychiatry as a keyword. The psychiatric areas covered include treating depression, mental illness, use of tricyclic anti-depressants and dosing SSRIs with specific cytochrome P450 genotypes. Incorporating pharmacogenomics into clinical decision support systems (CDSS) and translating knowledge into practice is challenging. These systems will help the clinician by transferring pharmacogenomics data to other health care providers and by informing physicians of patient’s results enabling them to request consultations based on the patient’s genotype^37^.

The best-known function of the cytochrome P450 enzymes is metabolism of drugs followed by the breakdown of toxic compounds including metabolic byproducts such as bilirubin which can lead to harmful results if not deactivated at a normal rate^2^. Natural variation in the activity of these enzymes can contribute to a range of drug responses and side effects on an individual basis. As these enzymes are encoded by genes with the wild-type allele occurring in most individuals, a normal or extensive metabolizer receives or inherits two copies of the normal wild-type allele from each parent and thus respond normally to drugs at standard doses. The doses initially established for a drug in the general population would depend on the specific cytochrome P450 genetic pattern. If an individual carries a loss-of-function allele along with the normal or wild-type allele (i.e., heterozygous state), then the enzyme coded by that gene would show reduced activity. If an individual carries two loss-of-function allele variants (i.e., homozygous or heterozygous state) such as two alleles showing deletion (i.e., *CYP2D6* *5) or non-functional pattern (i.e., *CYP2D6* *4), then a non-functional enzyme or an enzyme with reduced function would be labelled as a poor metabolizer for specific drugs degraded by that enzyme. If the individual carries duplications/multiplications of functional wild-type alleles, then the activity would be significantly increased and labeled as a rapid or ultra-rapid metabolizer. For example, homozygous and heterozygous carriers of *CYP2C9* gene variant alleles with reduced or no enzyme activity such as deletions would then require lower phenytoin doses to maintain therapeutic levels and to avoid an increased risk of toxicity with high drug doses for that patient prescribed for this anti-seizure drug^2,17^. Also, poor *CYP2C9* metabolizers with involvement of several anti-depressants lead to increased risk of adverse effects and lower doses are recommended for both fluoxetine and sertraline^8^. Individuals who inherit several copies (duplications) of the wild-type allele are labeled as rapid or ultra-rapid metabolizers and degrade specific drugs more quickly targeted by a particular enzyme and less effective in treating the illness. Increased toxicity is noted to occur in poor metabolizers with several psychotropics such as desipramine, haloperidol, amitriptyline and venlafaxine with *CYP2D6* variants influencing analgesic response to opioids such as codeine^8^. Physicians are increasingly prescribing the use of opioids to treat chronic pain and one of the most common reasons for patients seeking medical attention. Opioids and other analgesics are widely used in the clinic setting but can be problematic and potentially lethal. Twin studies have shown that opioid addiction has an inherited component of approximately 50% supported by genome-wide association study (GWAS) reports with gene variants having additive small effects including potassium ion channels (*KCNC1 and KCNG2*), a glutamate receptor related protein (*CNIH3*) and mu opioid receptor (*OPRM1)*^38^. Medications such as methadone to combat opioid addictions are regulated by cytochrome P450 genotypes (e.g., *CYP2B6*) and can also impact treatment response to addiction. Large clinical trials comparing effective pharmacotherapy for opioid addiction are needed including the role of pharmacogenetics.

## Clinical Trials and Gene Variants in Psychiatric Care

Studies of other genes not involving the cytochrome P450 enzymes have been reported to influence treatment of patients with major depression including inflammatory related genes such as IL-6 particularly in response to duloxetine^39^. Response to lithium treatment and bipolar disorder using GWAS data also linked two genes on chromosome 21 meeting significance criteria for an association with lithium response and warrants further study^40^. *TPH2* gene polymorphisms were also found to be associated with higher reduction rates in depressive symptoms in children treated with fluoxetine^41^ indicating genetic variability related to the brain serotonergic system seemingly associated with higher clinical improvement.

Risperidone is the most commonly prescribed antipsychotic medication in the US used to treat schizophrenia, bipolar disorder, severe dementia and irritability^42^. Risperidone is metabolized to an active metabolite by cytochrome P450 enzyme system, particularly *CYP2D6.* Individuals classified as poor metabolitizers may have a decreased capacity to metabolize risperidone and at a higher risk for adverse effects and those who are ultra-rapid metabolizes may have a decreased response to therapy. In addition, side effects of SSRIs found in older adults including sleep and sexual disturbances, dry mouth and diarrhea are associated with polymorphisms of the promoters of the serotonin transporter (*5-HTTLPR*) and *HTR1A* and *HTR2A* receptors influencing the functional status impacting on personalized patient care^43^.

## Conclusions and Commentary

Personalized medicine utilizing genetic fingerprints or molecular signatures to predict individual responses to drugs regarding efficacy, safety and pharmacokinetics is a growing area now entertained in clinical trials, development of drugs and study design with decisions for allocation of resources for clinical investigations including the selection of participants. Genotyping selected groups of individuals at risk for adverse events may allow possible reintroduction of affected drugs that were withdrawn at one time by regulators because of rare but serious adverse events. This rescuing or repurposing of drugs may become possible using next generation sequencing with development of disease specific pharmacogenetics testing in the areas of cardiology, hematology, endocrinology and psychiatry with interpretive services available depending on the testing results that may impact treatment, drug selection and dosage^5,18,44,45^. The interpretation of genetic patterns is increasingly important for clinicians not familiar with this new technology or maybe apprehensive to use the technology and personalized medicine approaches regarding their medical practice and sometimes driven by patients undergoing direct to consumer testing. This technology may also facilitate development of new drugs for vulnerable populations, rare diseases or to reduce ethnic disparities to improve public health.

Pharmacogenetics testing used to optimize treatment may be particularly important in mental health where 20% of the 121 pharmacogenetics markers are recognized by the FDA as informative for clinical practice involving psychiatric drugs^46^. This practice may positively impact financial and personal costs due to adverse drug events and save time in the course of selecting the most appropriate drug (e.g., psychotropic) and dosage for patients (e.g., in the field of child psychiatry). Reduced healthcare costs may occur by decreasing the number of adverse drug reactions, emergency visits and medications prescribed in order to yield effective therapies. Using this approach to identify whether an individual has a normal (extensive), rapid, or poor metabolizer status before a specific drug is prescribed should allow the practitioner to be more selective with a greater assurance of success based on an individual’s genetic pattern and therefore be more proactive. Because of the importance of inducers and inhibitors playing a role in medication management regarding the specific cytochrome P450 gene status either with other prescribed, over the counter drugs or dietary sources and their interaction, a careful history is required. For example, omega-3 fatty acid (eicosapentaenoic acid - EPA), a dietary supplement, acts as an inhibitor for *CYP2D6*^47^. Better awareness should be beneficial for both the patient and clinician but more investigations and clinical trials are needed to support this assumption in psychiatric care and treatment. The cost of genetic testing has also decreased through advances in technology and competition in spite of a growing number of analytes available with a faster turn-around time. The ease of testing with specimen collection (e.g., saliva instead of blood) and increasing recognition of medical necessity by third party payers will allow the process to move forward more quickly.

The growing list of personalized testing measures besides cytochrome P450 enzyme genes often includes other genes that play a role in the field of psychiatry such as neurotransmitter receptors (e.g., *5HT2C, DRD2*), transporters (e.g., *SLC6A4*), metabolic enzymes (*COMT*, *MTHFR*) and ion channel functions (e.g., *CACNA1C, ANK3*); all playing a role in pharmacodynamic aspects of psychiatric medications^18,44,48–58^. In addition, there is an association with anti-psychotic drug induced weight gain with a *5HT2C* receptor gene polymorphism^59^. Twelve SNPs representing eight other genes (*ADRAZA, ADRB3, BDNF, DRD2, GNB3, INSIG2, MC4R* and *SNAP25*) are also significantly associated with anti-psychotic-related weight gain such as risperidone^60^. An example on how personalized medical approaches can be used in the clinical setting was reported by Smith et al.^18^ and illustrated in a clinical case of an adolescent male requiring psychiatric services where recognition of pharmacogenetics testing results identified the potential source of his lack of tolerance for several medications.

Many genes are involved in mental illness with a rapidly expanding list of known clinically relevant or susceptible genes. For example, 560 genes are thought to play a role in schizophrenia;^61^ 390 genes for bipolar disorder^62^ and over 800 genes for autism spectrum disorder (ASD)^63^. Gene analytics and profiling programs have also shown that certain psychiatric or mental illnesses do overlap with disturbed gene networks such as circadian rhythm in schizophrenia, bipolar disorder and ASD along with calcium channel deficiencies^45^. In summary, it will be important to identify and characterize gene variants (existing and new) involved in mental illness and treatment response related to pharmacogenetics with the potential to lead to a better quality of life for those affected individuals. As an emerging field in medical care, pharmacogenetics testing and availability of coverage by insurance companies, availability of genetic services and local consultation expertise may vary from region to region nationwide. However, patients without an anticipated response to medications and those on multiple medications become candidates for pharmacogenetics testing. In addition, psychiatrists and psychologists can use their unique knowledge-base and experience with mental illness and aberrant behavior in the context of pharmacogenetics to help patients and clinicians make better decisions and choices regarding treatment plans and selection of medication as well as to educate other members of the healthcare provider team.

## Figures and Tables

**Table 1.** Cytochrome P450 (CYP) Enzymes and Drugs/Substrate Interaction.

Drug or Substrate	CYP 1A2	CYP 3A4/5	CYP 2B6	CYP 2C9	CYP 2C19	CYP 2D6	Drug or Substrate	CYP 1A2	CYP 3A4/5	CYP 2B6	CYP 2C9	CYP 2C19	CYP 2D6
alprazolam		M					modafinil		M				
amitriptyline	m	m!		m	M	M!	montelukast		M		M		
aripiprazole		M				M	naproxen	m			M		
atorvastatin		M					olanzapine	M					m
bupropion			M!				omeprazole		m			M	
caffeine	M	m					ondansetron	M	M				m
carbamazepine	m	M!		m			oxycodone		M				m!
celecoxib		m		M			paroxetine						M
citalopram		M			M	m	phenytoin				M	m	
clomipramine	M	M!			M!	m!	prednisolone		M				
clonazepam		M					propofol			M	m		
clozapine	M	M			m	m	propranolol	m				m	M
codeine		M			m!		quinine		M				
cortisol		M					ranitidine	m				m	m
dextromethorphan		m				M	risperidone		m!				M!
diazepam		M			M		sertraline		m	M	m	m	m
ecstasy (MDMA)	m	m				M	sildenafil		M				
erlotinib	m	M					simvastatin		M				
erythromycin		M					tamoxifen		M		m		M!
ethanol	m	m			m		terbinafine	M	M		M	m	
fluoxetine		m		M	m	M!	testosterone		M				
fluvoxamine	m					M	theophyline	M					
haloperidol	m	M				M	trazadone		M				m
hydrocodone		M				m!	triazolam		M				
ibuprofen				M	m		trimipramin				m	m	M
imipramine	M	M			m	M	valproic acid				M!		
lidocaine	M	m					venlafaxin		m				M!
methadone	m	M	M		m	m	verapamil	m	M		m		
methyprednisolone		M					warfarin	m	m		M	m	
mirtazapin	m	M				M	zolmitriptan	M					

Major metabolizer = M

Produces active metabolite = !

Minor metabolizer = m

Modified from Samer et al.^8^

**Table 2.** Cytochrome P450 CYP1A2 Enzyme Substrates, Inhibitors and Inducers.

Cytochrome P450 Gene	Drugs or Substrates	Inhibitors	Inducers
CYP1A2	Agomelatine, Amitriptyline (Elavil®), Caffeine, Clomipramine (Anafranil®), Clozapine (Clozaril®), Cyclobenzaprine (Flexeril®), Erlotinib (Tarceva®), Estradiol, Fluvoxamine (Luvox®), Imipramine (Trofranil®), Haloperidol (Haldol®), Melatonin, Mexiletine (Mexitil®), Naproxen, Olanzapine (Zyprexa®), Ondansetron (Zofran®), Propranolol, Ropivacaine, (Naropin®), Tacrine (Cognex®), Theophyline (Theolair®), Verapamil (Covera-HS®), Warfarin (Coumadin®), Zolmitriptan (Zomig®)	Amiodarone (Cordarone®), Cimetidine (Tagamet®), Ciprofloxacin (Cipro®), Cumin, Fluroquinolones, Fluvoxamine (Luvox®), Interferon, Methoxsalen (Uvadex®), Milbefradil	Beta-Naphthoflavone, Broccoli, Brussel sprouts, Cabbage Cauliflower Insulin, Methylcholanthrene, Modafinil (Provigil®), Nafcillin, Omeprazole (Prilosec®), Tobacco
